# Comparing high-intensity resistance training incorporating periodic eccentric-only exercise with blood flow restriction training in older men: physiological responses and exercise feasibility

**DOI:** 10.1007/s00421-026-06452-x

**Published:** 2026-09-24

**Authors:** Wouter A. J. Vints, Lina Mickevičienė, Orgesa Qipo, Ivan Bautmans, Artūras Sujeta, Dovilė Kielė, Mantas Mickevičius, Sigitas Kamandulis, Vida J. Česnaitienė, Oron Levin, Nerijus Masiulis

**Affiliations:** 1 Department of Health Promotion and Rehabilitation, Lithuanian Sports University, Sporto Str. 6, 44221 Kaunas, Lithuania; 2 Department of Rehabilitation Medicine Research School CAPHRI, Maastricht University, Maastricht, The Netherlands; 3 Centre of Expertise in Rehabilitation and Audiology, Adelante Zorggroep, Hoensbroek, The Netherlands; 4 Gerontology Department, Vrije Universiteit Brussel (VUB), Laarbeeklaan 103, 1090 Brussels, Belgium; 5 Frailty and Resilience in Ageing (FRIA) Research Unit, Vitality Research Group, Vrije Universiteit Brussel (VUB), Laarbeeklaan 103, 1090 Brussels, Belgium; 6 Geriatrics Department, Universitair Ziekenhuis Brussel, Laarbeeklaan 101, 1090 Brussels, Belgium; 7 SOMT University of Physiotherapy, Softwareweg 5, 3821 BN Amersfoort, The Netherlands; 8 Educational Research Institute, Vytautas Magnus University, 44248 Kaunas, Lithuania; 9 Product Development Manager, Nature Labs, UAB, 01402 Vilnius, Lithuania

**Keywords:** Blood flow restriction training, High-intensity resistance training, Eccentric exercise, Older adults, Exercise-induced muscle damage

## Abstract

**Aim:**

This study aimed to compare the feasibility, strength adaptations, and physiological effects of high-intensity resistance training incorporating periodic eccentric-only exercise (HIRT) with blood-flow restriction training (BFRT) in older men.

**Methods:**

This three-group randomized controlled trial included 63 older men assigned to lower limb HIRT, BFRT, or a non-exercising control group for 12 weeks. BFRT was at 40% one-repetition maximum (1RM) and HIRT altered between eccentric-only exercises at 120% 1RM and traditional resistance exercise at 80% 1RM in a 1:3 ratio. Outcome measures included ratings of perceived exertion (RPE), delayed onset muscle soreness (DOMS), lactate and creatine kinase (CK) levels, 1RM and adverse events.

**Results:**

BFRT was associated with higher RPE and more frequent training till failure, as well as greater lactate responses. Eccentric sessions in HIRT resulted in higher DOMS and CK levels. Both interventions significantly improved 1RM compared to controls, with HIRT demonstrating greater strength gains in leg extension and leg press. Adverse events included elevated blood pressure (n=1) and headache (n=2) in BFRT, and knee pain (n=2) in HIRT.

**Conclusion:**

Both interventions were effective but imposed different physiological demands, with BFRT inducing greater metabolic and HIRT greater mechanical stress. HIRT led to slightly greater strength gains, while BFRT remains a viable alternative for those unable to perform high-load exercise. Incorporating eccentric exercise may further optimize adaptations and manage muscle damage.

**Supplementary Information:**

The online version contains supplementary material available at https://doi.org/10.1007/s00421-026-06452-x.

## Introduction

Age-related decreases in muscle mass and strength, particularly of the lower limbs, are strongly associated with physical disability, poor quality of life and death (Santilli et al. 2014; Herold et al. 2019). Physical activity guidelines for older adults recommend at least two days of resistance exercise training per week (WHO Guidelines on Physical Activity and Sedentary Behaviour 2020). The most recommended strategy to counteract age-related loss of muscle mass and strength is high-intensity resistance training (HIRT), i.e. lifting loads above 70% of the one repetition maximum (1RM) (Fragala et al. 2019; García-Pinillos et al. 2019; Lichtenberg et al. 2019). Specifically the integration of eccentric or eccentric-only exercises (EOE) into HIRT programs has proven particularly beneficial for older adults (Quinlan et al. 2019; Harris-Love et al. 2021) as eccentric movements play a crucial role in older adults’ daily activities, such as descending stairs or sitting down in a chair (Cvečka et al. 2023). During eccentric training, muscle fibres can be subjected to higher mechanical loads than during regular resistance exercise (RE). This mechanical stimulus leads to a significant increase in muscle strength (Crewther et al. 2005). Although eccentric training is generally associated with lower ratings of perceived exertion (RPE) than traditional RE (Hollander et al. 2003), it is associated with greater muscle damage, especially in persons unaccustomed to this type of training, regardless of initial muscle condition or age (Lovering & Brooks 2014). The manifestation of this damage is characterised by the presence of muscle pain, reduced muscle strength and elevated blood creatine kinase (CK) levels 24–48 h after training (Nikolaidis et al. 2008). However, thanks to muscular adaptations pain and muscle damage during and following subsequent exercise bouts are reduced, which is referred to as a repeated bout effect (RBE) (McHugh 2003). Still, some authors advocate that eccentric training is overly demanding for (the muscles of) older adults, and the high training intensity is potentially discouraging participation, hindering their long-term commitment to exercise (Lovering and Brooks 2014; Gault and Willems 2013). Hence, there remains a lack of consensus whether eccentric training is feasible in older adults.

Alternatively, low-load resistance training, performed at 40% of estimated 1RM in the present study, combined with blood flow restriction has been proposed as an effective method to enhance muscle strength and hypertrophy in older adults and in individuals who are unable to engage in high-intensity exercise or for whom HIRT is not feasible (Yuan et al. 2023). Blood flow restriction training (BFRT), otherwise referred to as occlusion training or KAATSU training, involves the in-exercise application of external pressure on human limbs using a pneumatic cuff or elastic band (Abe et al. 2006). This pressure restricts venous return from the limbs and partially reduces arterial inflow, thereby inducing muscle hypoxia (Patterson et al. 2019) and increasing plasma lactate concentration during low-intensity resistance exercise (Takarada et al. 2000). In contrast to HIRT, the muscular strength and hypertrophy gains promoted by BFRT are predominantly achieved through a metabolic training stimulus, rather than a mechanical one (Kon et al. 2012; Pearson and Hussain 2015). While BFRT can induce a training stimulus with lower weights compared to conventional RE, it is suggested that the former is associated with heightened levels of discomfort and perception of fatigue in comparison to the latter, due to the rapid accumulation of metabolites such as lactate. This may also lead to a reduced adherence to such exercise programme (Spitz et al. 2022).

Overall, it remains unclear whether HIRT, comprising periodic EOE sessions interspersed with conventional resistance exercise (RE), and BFRT are feasible training methods for older adults, particularly in relation to safety, exercise-induced muscle damage, and perceived exertion. Therefore, our first aim was to report participants’ training adherence and rate of adverse events for HIRT and BFRT. Second, we aimed to characterize delayed-onset muscle soreness and creatine kinase responses following repeated high-load EOE sessions over the 12-week intervention in older men. The HIRT protocol was informed by Margaritelis et al. (2015), who showed that alternating eccentric-only and concentric-only exercise could attenuate the RBE in young adults. Third, we aimed to compare subjective ratings of muscle pain and RPE, and objective levels of CK and lactate between both resistance training conditions. By evaluating these two different training modalities in older men, we aimed to determine the feasibility and confirm the differences in training stimulus of two mechanistically different training modalities suggested to mitigate sarcopenia and improve physical function in an ageing population.

## Methods

### Pre-registration

This study is part of a trial named Blood Flow Restriction and High-intense Resistance Training in Aging: Interactions Between Neuroplasticity and Muscle (BRAIN-M), registered in ClinicalTrials.gov under Identifier number NCT05744167. Data from this project related to muscle–tendon responses to training have been published before (Cesanelli et al. 2026).

### Participants and setting

Between October 2022 and November 2023, sixty-three men aged between 60 and 78 years old were recruited in the BRAIN-M trial conducted at the Lithuanian Sports University, Kaunas, Lithuania. Recruitment strategies were the use of advertisements in local and national newspapers, on events organized for older persons, and the university website. A participant flow diagram is presented in Supplementary Figure S1.

Next to age and sex, additional exclusion criteria were alcohol or drug abuse, cognitive, neurologic, cardiovascular, oncologic or psychiatric disorders, previous lower extremity injury, osteoarthritis, diabetes mellitus, obesity (body mass index [BMI] above 30 kg/m^2^), history of deep vein thrombosis, use of psychopharmacological drugs in the last five years, or a history of chemotherapy. This information was collected from information the participants provided, either subjective information or from provided medical documents. Finally, we excluded subjects that participated on a regular basis in an exercise program in the six months preceding enrolment. Participants were allowed to withdraw from the study at any time. The study methods were approved by the Kaunas Regional Biomedical Research Ethics Committee (No. 2022-BE−10-0012) and a written informed consent was obtained from all participants prior to their inclusion in the study.

### Study design

This study was a parallel 3-group randomized-controlled trial comparing two different resistance exercise interventions and a control condition. Group allocation was done by minimization, a method of ensuring balance between the groups for one or more prognostic factors (Taves 1974); In the BRAIN-M trial, handgrip strength at baseline (in kg) was considered a variable with important prognostic value and was therefore entered in the minimization method as a continuous variable, keeping the differences in handgrip strength at baseline as small as possible between groups. The minimization program was built by author WV in Excel. The highest handgrip value measured from 4 trials (two per hand) was entered in the minimization program. The minimization procedure was as follows: The first six participants were included completely at random in one of the three groups. Subsequently, any new participant was included in a group according to the following rules: (1) with the chosen group allocation, the sum of the differences between mean handgrip strength in each of the groups is the lowest and (2) the number of participants in the largest group is not more than three participants larger than the number of participants in the smallest group. In case a participant would have to enter the largest group according to the first rule, making this group more than three participants larger than the smallest group, the second best option according to the first rule was chosen instead. The second rule accounts for the potential situation that only few participants are entered in one of the groups and at the end of the project all remaining participants have to be enrolled in the same group, which possibly results in large differences in handgrip strength in this group compared to the other two groups. Participants and coaches were aware of the group allocation, but group allocation was blinded for anyone who performed assessments or analysis of the results, except for RPE measurements which were performed by the coaches. Participants age, educational level (in years), and smoking status were recorded. Height and weight were measured, and body mass index (BMI) was calculated as weight in kilograms divided by height in meters squared (kg/m^2^).

Physical activity level was estimated using the International Physical Activity Questionnaire – Short Form (IPAQ-SF). Self-reported physical activity level was calculated based on the total kcal burned per week during exercise of light, moderate or vigorous intensity, using the formula: total kcal/week = the sum of days performing light/moderate/vigorous activity × average time/day performing these activities × F, where F equals 3.3 for light intense exercise, 4.0 for moderate intense exercise and 8.0 for vigorous intense exercise. Participants burning less than 600 kcal per week were categorized as sedentary, 600–3000 kcal per week as moderately active and > 3000 kcal per week as highly active (Committee 2005).

### Assessment: strength measurements

Baseline strength was expressed as the estimated one-repetition maximum (1RM) for each of the three exercises included in the intervention: the leg press, leg extension, and leg curl. A 5RM test was performed using a standard protocol recommended by the National Strength and Conditioning Association (Haff and Triplett 2015). Following familiarization and a warm-up, the load was progressively increased across successive attempts. Participants rested for 2–4 min between attempts and were asked whether they felt sufficiently recovered before continuing. After each attempt, they were asked how many additional repetitions they believed they could perform. When a participant completed five repetitions with correct technique and reported being unable to perform another repetition, the corresponding load was recorded as the 5RM. The estimated 1RM was subsequently derived from the 5RM load using the ExRx.net calculator (Swain and Brawner 2014). (ExRx.Net: Predicting One-Rep Max 2026). The 5RM test was repeated every four weeks in both experimental groups to adjust training loads according to changes in strength and maintain the prescribed relative training intensity. The 5RM test was repeated in all three groups after completion of the 12-week intervention.

Handgrip strength (kg) was measured in a standing position using a mechanical spring-type Saehan Smedley hand dynamometer (Saehan Corporation, Republic of Korea; product reference 08–010401). The grip width was individually adjusted according to hand size to allow a comfortable grip and maximal force production, and the indicator was reset to zero before each trial. Participants first performed one submaximal familiarization trial, followed by two maximal effort trials with each hand. The hands were tested alternately, allowing a 1 min rest period between trials performed with the same hand. The highest value obtained across the four maximal trials was used for analysis.

### Assessment: blood analysis

After 15 min of seated rest, capillary lactate levels were measured with the Arkray Lactate Pro 2 device from a drop of blood collected on a test strip after pin-prick in a fingertip. Lactate measurements were done before and immediately after the first exercise session. CK levels were measured from whole blood collected at the antecubital vein in an EDTA tube before and 48 h after the first exercise session (i.e. before the second training session of that week). CK measurements 48 h after exercise were repeated once every 4 weeks, i.e. 48 h after every EOE session in the HIRT group (see Supplementary Table S1).

### Intervention: resistance exercise intervention

Participants in the BFRT and HIRT exercise groups trained two times per week for a total of 12 weeks. The training session started each time with a 10 min warm-up session, consisting of cycling on a cycle-ergometer at a power (W) equal to their own body weight in kg and 60–80 revolutions per minute. This was followed by a resistance exercise intervention, consisting of three lower limb exercises, specifically leg press, leg extension and leg curl, lasting in total 40–50 min. Supplementary Table S1 illustrates the characteristics of both RT interventions and their progressions schematically. Both exercise interventions were made equal in their relative volume (V% = number of repetitions × number of sets × number of exercises × %1RM) = 57.6%. Training loads were prescribed using estimated 1RM values derived from the 5RM test, as described in the Strength measurements subsection. *BFRT* was performed at a relative intensity of 40% of 1RM with an occlusive cuff placed at the proximal end of both lower extremities to restrict the venous return of blood flow. Blood flow restriction was applied using the SmartCuffs 3.0 PRO Elite system (Smart Tools Plus LLC, Strongsville, OH, USA). Four-inch (approximately 10 cm) pneumatic cuffs were placed proximally on both thighs. Individual ALOP was determined automatically by the device before training. The cuffs were inflated to 50% of the individual ALOP during the first week. Pressure subsequently increased by 10 percentage points each week until reaching 80% of ALOP in week 4, which was maintained for the remainder of the intervention. The time under tension was 1–2 s per repetition. BFRT exercises were performed for 4 sets of 12 repetitions with a 30-s rest period between sets and 3 min rest between exercises. During the 3 min rest period, participants were allowed to deflate the occlusive cuffs. The HIRT protocol followed a repeating four-session cycle comprising one EOE session at 120% of the estimated 1RM followed by three conventional RE sessions at 80% of the estimated 1RM (EOE:RE ratio of 1:3). During an EOE training session, the concentric phase of the movement was completely supported by two coaches. The eccentric phase of the movement was accentuated by increasing the time under tension to 5 s per repetition. Each exercise was performed for 4 sets of 4 repetitions with 2 min rest between sets and 3 min rest between exercises. During RE, both the concentric and eccentric phase of the movement were unsupported (see Supplementary Figure S2). The time under tension was 1–2 s per repetition. Each exercise was performed for 4 sets of 6 repetitions with 2 min rest between sets and 3 min rest between exercises. Missed exercise sessions were compensated for by adding a session at the end of the protocol.

### Exercise effort characteristics

At the end of each set of each of the three exercises (i.e. twelve times per training session), participants were inquired about their RPE on a 10-point Borg scale (Morishita et al. 2019). Higher scores indicated a higher RPE. For between-group comparisons, the following variables were calculated: (1) mean RPE of the first session; (2) mean RPE across the entire program excluding the first session; (3) mean RPE of the last set of each exercise across the entire program excluding the first session; and (4) total number of completed sets. Participants’ RPE measurements were not used for statistical analysis if more than 50% of the values to calculate one of these means were missing (due to drop-out of the participant, n = 9, or not recording, n = 9).

It was recorded if the requested amount of reps in a set could not be completed due to muscle failure. In case the last set was not performed due to muscle failure upon the second to last set, this value was left blank for the mean RPE of the whole program calculation, and the maximum value (10/10) was entered for the calculation of the mean RPE of the last set.

**Fig. 1:**
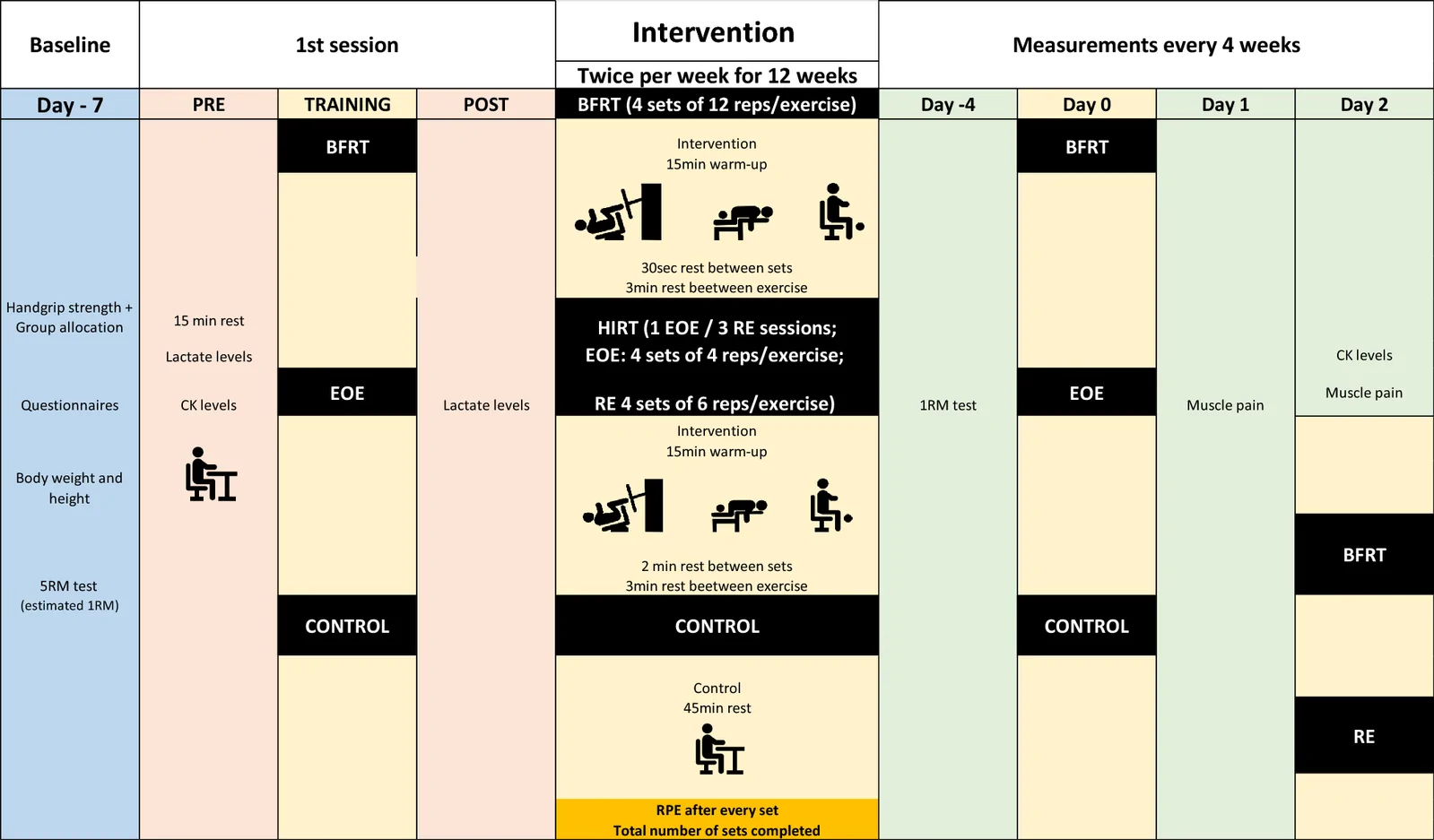
Overview of the study design and measurement timeline. Participants were allocated to BFRT, HIRT, or control groups and followed a 12-week intervention protocol. The figure illustrates the timing of strength assessments, blood sampling (lactate and creatine kinase), perceived exertion (RPE), and muscle pain (DOMS) measurements, as well as the structure of the exercise sessions

Furthermore, muscle pain was recorded on a visual analogue 10-point scale (VAS) 24 and 48 h after every training session, representing delayed-onset muscle soreness (DOMS). Higher scores indicated more pain. An overview of all measurements is illustrated in Fig. 1.

### Feasibility criteria for exercise interventions

The feasibility of the BFRT and HIRT intervention for application in older adults was assessed based on set completions and safety criteria. Set completion rate was calculated as the total number of sets completed for all participants that started the intervention divided by the total number of intended sets (excluding drop-outs during the baseline assessments, but including drop-outs during intervention). A set completion rate ≥90% was categorized as good, 80–89% as acceptable, 60–79% as borderline (requiring adaptation), and <60% as not feasible as designed. These thresholds do not represent universally validated cut-offs. Furthermore, adverse events were recorded and when needed a medical professional was consulted to decide the relationship of the adverse event and the intervention and decide on continuation of the study protocol or drop-out of the participants. Any adverse event was reported, but participants were specifically asked about pain (tendomuscular, joint pain or headache), and any symptoms related to cardiovascular changes (dizziness, fainting, blurred vision). Blood pressure and heart rate were recorded at baseline and measured again in case of symptoms.

### Statistical analysis

IBM SPSS Statistics version 27 (IBM Inc., Chicago, USA) was used to perform all analyses. First, the data was inspected for outliers and normality. Extreme outliers, defined as values lying more than 3× the interquartile range away from the median were excluded. Normality was defined as a kurtosis and skewness measure between − 2 and +2. Additionally, normality was checked visually using PP-plots and histograms. Data not meeting one of the normality assumptions was log transformed. Homoscedasticity was tested with the Levene’s test. Group differences between the three groups in baseline descriptives were analysed using one-way ANOVA. For comparison between two groups, independent t-tests and Chi^2^ tests (or Fisher Exact tests, if the expected count in any of the cells was below 5) were used for continuous and categorical variables, respectively. Repeated measures ANOVA was used to evaluate the changes in 1RM results between groups over time. Data visualization was done in JASP (Version 0.19.3.0).

## Results

### Participant characteristics

Sixty-three older men (age range: 60–78) with a BMI ranging between 21.3 kg/m^2^ and 32.9 kg/m^2^ and maximum handgrip strength ranging between 34 and 60 kg were included. Table 1 includes the baseline characteristics. There were no baseline differences between groups, except for baseline 1RM values on the leg press (mean BFRT group: 170.74 (33.31) kg, mean HIRT group: 206.21 (41.17) kg, independent t-test p = 0.012).

**Table 1:** Baseline characteristics

	BFRT (n=21)	HIRT (n=21)	CON (n=21)	Total (n=63)	p value
Age	67.19 (5.17)	66.71 (3.44)	65.05 (4.47)	66.31 (4.44)	0.264
Handgrip strength (kg)	48.07 (5.59)	47.79 (6.03)	47.64 (5.24)	47.83 (5.54)	0.969
BMI (kg/m^2^)	26.40 (2.88)	26.40 (3.01)	26.22 (2.92)	26.34 (2.89)	0.384
Educational level					0.971
Higher education	18 (85.71%)	19 (90.48%)	16 (84.21%)	53 (86.89%)	
Secondary education	1 (4.76%)	1 (4.76%)	1 (5.26%)	3 (4.92%)	
Basic education	2 (9.52%)	1 (4.76%)	2 (10.53%)	5 (8.20%)	
IPAQ kcal burned/week	5816.50 (5252.02)	5678.71 (6253.97)	4684.50 (4216.62)	5422.10 (5289.67)	0.781
Physical activity level					0.639
Sedentary	0 (0%)	1 (4.76%)	1 (5.56%)	2 (3.39%)	
Moderately active	7 (35%)	10 (47.61%)	9 (50.00%)	26 (44.07%)	
Highly active	13 (65%)	10 (47.61%)	8 (44.44%)	31 (52.54%)	
CK (μkat/L)	1.77 (1.01)	1.71 (0.99)	2.19 (1.36)	1.88 (1.12)	0.384
Lactate (mmol/L)	2.22 (1.06)	2.14 (1.09)	2.10 (0.95)	2.16 (1.02)	0.930
1RM leg extension (kg)	72.34 (14.25)	71.02 (18.99)	71.33 (17.78)	71.57 (16.74)	0.975
1RM leg curl (kg)	42.21 (9.51)	44.71 (10.08)	46.47 (13.71)	44.43 (11.07)	0.570
1RM leg press (kg)	170.74 (33.31)	206.21 (41.17)	198.07 (43.95)	191.54 (41.74)	0.039*

*BFRT* blood-flow restriction training, *BMI* body mass index, *HIRT* high-intensity resistance training, *IPAQ* international physical activity questionnaire

*p < 0.05 for ANOVA test result. Values are presented as mean (SD) or n (%)

### Exercise effort characteristics and adherence

Both training interventions were substantially demanding for the participants based on RPE, missed sets and DOMS recordings, see Table 2. However, some notable differences between the training groups regarding effort existed. Adherence not excluding drop-outs during intervention was acceptable (84.81%) in the BFRT group and good (93.99%) in the HIRT group. In the BFRT group, participants that were still continuing exercise training were not able to complete all four sets of an exercise in 142 occasions, while in the HIRT group only 4 sets were skipped across all the participants and across all training sessions. Additionally, during the first training session, mean RPE score per set was higher in the BFRT group compared to the HIRT group (p=0.005). The mean RPE score across all sessions (first session excluded) of the last set was also significantly higher in the BFRT group (p < 0.001). Mean lactate levels increased from pre- to post-exercise in both the BFRT and HIRT groups during the first exercise session (both p < 0.001; see Supplementary Table S2). However, this increase in lactate levels was significantly higher in the BFRT compared to the HIRT group (p = 0.010). Furthermore, mean DOMS scores 24 h and 48 h after the first training session did not significantly differ between both resistance exercise groups, and post-exercise VAS scores were on average very low, yet significantly higher in the HIRT group compared to the BFRT group, 24 h after all the exercise sessions (first session excluded, p=0.034). Regarding the mean DOMS scores following EOE sessions (i.e. week 4, 8, and 12; first session excluded), the mean DOMS scores were slightly more elevated, both after 24 h (VAS=1.86 (1.61)) and after 48 h (VAS=1.21 (1.24)). Moreover, mean CK levels in the HIRT group, but not in the BFRT group, were significantly increased 48 h after the first exercise session and 48 h after EOE sessions in week 4, 8 and 12, compared to pre-intervention measurements (both p < 0.001, see Supplementary Table S2). There were large inter-individual differences among participants for DOMS scores and CK level responses, see also Supplementary Figure S2, yet the overall trend was that both DOMS and CK levels were decreased when comparing the average of week 4, 8 and 12 with the first exercise session, which is most likely reflects a RBE.

**Table 2:** Exercise effort characteristics and post-intervention 1RM comparing exercise groups

	BFRT	HIRT	Total	p-value
First session exercise effort				
Mean of RPE after every set	6.66 (1.66)	5.00 (1.47)	5.98 (1.76)	0.005*
Mean of RPE of last set/exercise	8.28 (1.80)	5.31 (1.45)	7.06 (2.22)	<0.001*
Post lactate levels (mmol/L)	7.08 (2.81)	4.55 (3.09)	5.88 (3.18)	0.010*
CK levels 48 h after first session (μkat/L)	4.47 (3.20)	8.16 (10.63)	6.22 (7.79)	0.173
DOMS 24 h after first session	2.17 (2.32)	3.31 (2.59)	2.76 (2.49)	0.261
DOMS 48 h after first session	1.42 (1.56)	2.85 (2.70)	2.16 (2.30)	0.123
Chronic exercise effort				
Total number of sets not done due to muscle failure	142/5040 (2.82%)	4/4344 (0.09%)	146 (1.56%)	NA
Mean RPE after every set	6.40 (1.33)	5.61 (1.19)	6.01 (1.30)	0.136
Mean RPE of last set/exercise	8.45 (1.13)	5.80 (1.24)	7.13 (1.79)	<0.001*
Mean CK levels 48 h after w4, w8 and w12 (μkat/L)	2.47 (1.65)	3.10 (2.41)	2.78 (2.05)	0.362
After w4	2.76 (2.23)	3.22 (4.23)	3.00 (3.34)	
After w8	2.48 (1.73)	3.95 (3.35)	3.17 (2.68)	
After w12	2.29 (1.87)	2.61 (1.78)	2.45 (1.81)	
Mean DOMS 24 h after each session	0.27 (0.28)	1.03 (1.10)	0.72 (0.93)	0.034*
Mean DOMS 48 h after each session	0.16 (0.17)	0.70 (0.85)	0.48 (0.71)	0.051
Post-intervention 1RM				
1RM leg extension (kg)	84.54 (16.69)	98.28 (15.60)	91.41 (16.15)	0.023*
1RM leg curl (kg)	52.00 (7.15)	55.07 (7.71)	53.54 (7.44)	0.246
1RM leg press (kg)	238.89 (52.61)	286.31 (39.38)	262.60 (46.47)	0.007*

*BFRT* blood-flow restriction training, *DOMS* delayed-onset muscle soreness, *EOE* eccentric-only exercise, *HIRT* high-intensity resistance training, *NA* not applicable, *RPE* rate of perceived exertion, *VAS* visual analogue scale

*p < 0.05 for t-test results. Values are presented as mean (SD). RPE and DOMS were scored on 10-point numeric rating scales, with higher values indicating higher levels of perceived exertion or muscle pain, respectively. These values were not recorded for control group participants

### Strength improvements

**Fig. 2:**
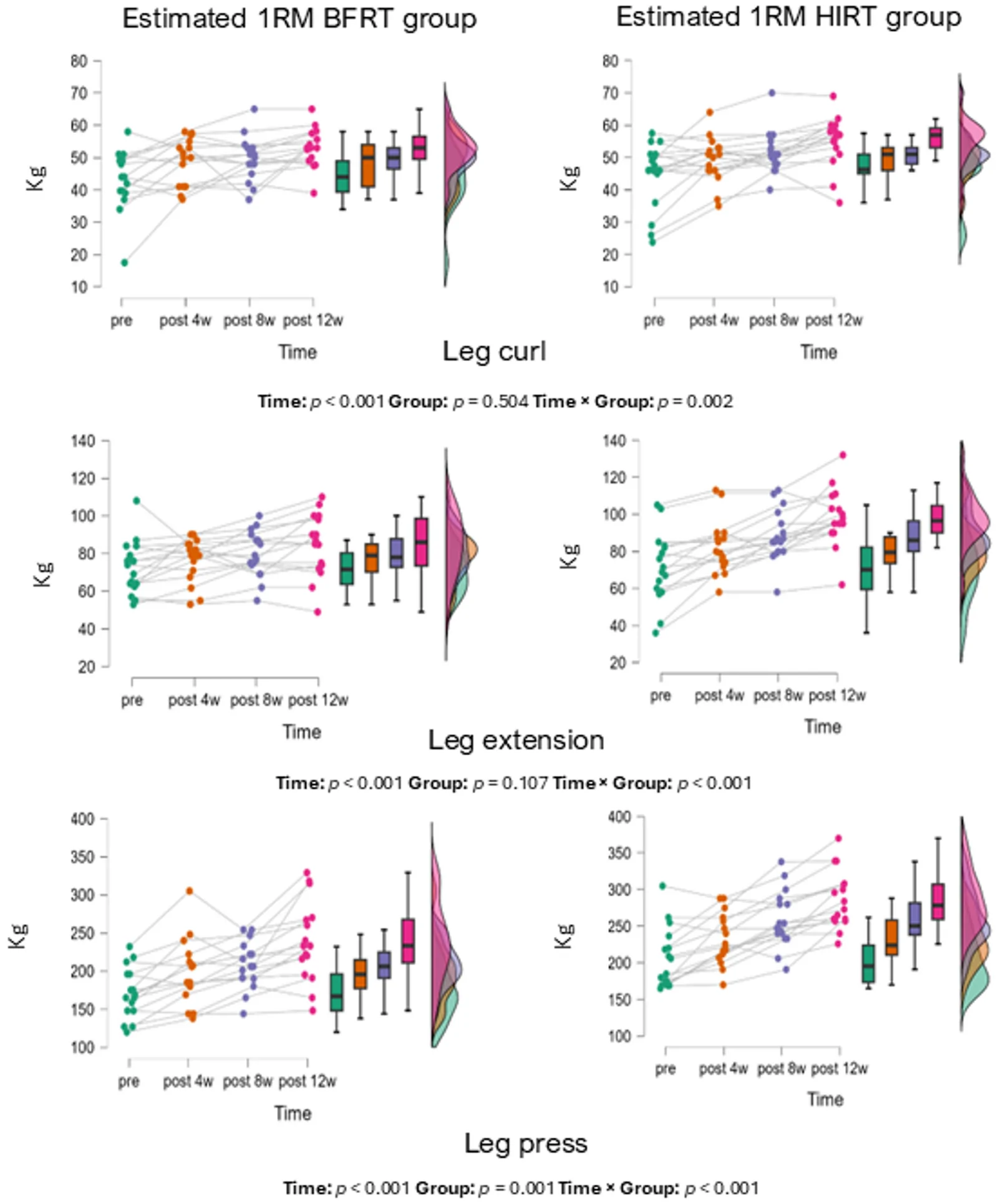
Changes in estimated one-repetition maximum (1RM) strength over time in the BFRT and HIRT groups. Individual values and group distributions are shown for leg curl, leg extension, and leg press exercises at baseline (pre), post 4 weeks, post 8 weeks, and post 12 weeks. Panels on the left represent the BFRT group, and panels on the right represent the HIRT group

Participants in both exercise groups significantly improved on their 1RM values over time for all three exercises (all p ≤ 0.001), while control group participants did not improve (individual values depicted in Fig. 2). Repeated measures ANOVA found a significant interaction effect for strength improvement on all of the 1RM tests (all p ≤ 0.002), see Supplementary Table S2. Comparing post-intervention means between exercise groups, participants in the HIRT group showed significantly larger improvements in 1RM on leg extension (p = 0.023) and leg press (p = 0.007) compared to participants in the BFRT group.

### Adverse events and reasons for drop-outs

In the BFRT group, one participant reported a self-detected increase in resting blood pressure after three weeks of training. As blood pressure was not routinely monitored as part of the study protocol, the participant sought medical evaluation independently. Following consultation with a cardiologist, discontinuation of the training intervention was recommended. Another participant suffered a leg injury unrelated to the training session after seven weeks of the intervention. He was unable to continue training. Furthermore, the study was discontinued by two participants due to the onset of headaches. Specifically, one person experienced a sudden jolt in the head and a severe headache during exercise, which disappeared after a few hours. The participant consulted a medical doctor, but no specific diagnosis was made. Concerned that the situation would recur, the participant decided to discontinue participation in the study. The other individual reported the onset of a headache following a training session that persisted for several hours. As this pain recurred after subsequent training sessions, the participant ceased training. One person withdrew from the study due to the onset of back pain. This individual suddenly experienced discomfort in the lower back during an exercise session, with symptoms persisting for a duration of several days. The participant expressed concern that the pain would reappear and withdrew from the study. Two participants from the HIRT group withdrew from the study due to chronic knee pain, which was aggravated by the training and linked to pre-existing knee issues. Additionally, another participant withdrew before starting training after MRI scanning revealed dilated blood vessels, requiring further clinical diagnosis. Finally, one participant in the HIRT group decided not to continue the pre-test assessments for personal reasons.

Six participants in the control group withdrew from the study for the following reasons: one participant fainted during blood collection and decided not to continue, two participants withdrew because they did not want to undergo MRI scanning, one participant had an MRI scan that showed a cyst in the brain, requiring further diagnostics, and one participants refused to continue pre-test assessments and one participant refused to return for post-test assessments without providing reasons.

Overall, adverse events were more frequent in the BFRT group and were primarily related to systemic responses (e.g., headache, blood pressure), whereas events in the HIRT group were mainly musculoskeletal and linked to pre-existing conditions.

## Discussion

The present study investigated the feasibility of two distinct RT modalities (HIRT with EOE and RE sessions, and BFRT) in older men, focusing on training adherence, reported adverse events, physiological responses, effort characteristics, and changes in 1RM. Given the potential benefits of these exercises for older adults, it is important to understand their applicability, tolerability and potential risks to ensure effective adaptation to this population.

As was aimed during the design of the project, the results of the study indicate that both training interventions were strenuous for the participants. However, some notable differences between the training groups regarding effort existed. In the BFRT group, participants were not able to complete all four sets of an exercise on 142 occasions. While more than half of the participants completed all sets, some participants repeatedly stopped an exercise at 3 out of 4 sets. It may have been caused by the high % of ALOP which is linked to RPE (Bell et al. 2018) or the increasing discomfort of the inflated cuffs around the lower limbs. In addition, six participants in the BFRT group withdrew from the study for various reasons, four of which were due to exercise-related adverse events, including increased resting blood pressure (n = 1), headache (n = 2), and lower back pain (n = 1). These events occurred in the context of resistance training and could not be specifically attributed to the application of blood flow restriction. However, none of the reported adverse events resulted in long-lasting effects, and all symptoms resolved without further complications. Previous literature suggests that BFRT can cause side effects such as headaches and high blood pressure. A survey conducted by Patterson and Brandner (2018) found that 15.6% of respondents expressed concern about adverse cardiovascular effects, including increased blood pressure, and 14.6% of professionals reported that their clients experienced headaches. These data are complemented by a narrative review by Nascimento et al., (2022), which suggests that sympathetic activation during BFRT may lead to an increase in blood pressure, especially in individuals prone to hypertension or other circulatory disorders, and that circulatory disturbances and changes in intracranial pressure may lead to headaches. Therefore, to minimize the potential adverse effects of BFRT, it is recommended that training should only be performed under expert supervision, that pressure settings should be tailored to each individual's ALOP, and blood pressure should be monitored before, during and after BFRT sessions, especially in individuals with cardiovascular risk factors (Nascimento et al. 2022; Patterson and Brandner 2018).

In the HIRT group only four sets were skipped by all participants across all training sessions. Four participants withdrew from the HIRT group, two because of exercise-induced knee pain. Both participants reported having a history of knee pain and informed the coaches that the high-intensity exercise exacerbated their existing problems. Previous literature has highlighted that resistance exercises with high loads can exacerbate knee pain in individuals with osteoarthritis, as excessive loading of the joint can promote inflammatory processes and increase discomfort (Tanaka et al. 2013). Tanaka et al. (2013) proposed that individuals suffering from knee osteoarthritis or a history of knee pain should refrain from excessive joint loading and instead prioritise strengthening exercises performed under reduced loading conditions in order to minimise pain and inflammation. This underscores the importance of individualized exercise prescription for those with pre-existing joint issues.

Regarding perceived exertion, the BFRT group demonstrated higher subjective training intensity compared with the HIRT group. Specifically, RPE values were significantly higher in the BFRT group during all sets of the initial training session (p = 0.005) and during the final set of each exercise across the entire training program (p < 0.001).This is in line with previous studies, which have indicated that BFRT elicits greater discomfort compared to conventional RT (Bell et al. 2018). One factor that may contribute to a higher RPE is an increase in lactate. Our study demonstrated that the BFRT group exhibited a significantly higher increase in lactate compared to the HIRT group (p=0.010), which may influence the level of discomfort (Takarada et al. 2000). Furthermore, Bell et al. 2018 demonstrated that elevated circulatory restriction pressure (80% of the ALOP) was a significant factor in increasing RPE scores. Given that 80% of the ALOP was also applied in our study, this may provide a rationale for why participants in the BFRT group experienced such high subjective strain. Hence, while high percentages of ALOP has been recommended in order to provide a more effective muscle stimulation (Rolnick et al. 2021), we recognize it can also have a detrimental effect on exercise tolerance and motivation. This is in line with the recommendation of Spitz et al. (2022), who suggested that lowering the percentage of ALOP may reduce discomfort and improve adaptation to BFRT without sacrificing performance. Further research should investigate whether training at lower percentages of ALOP can sustain the advantages of BFRT whilst concomitantly enhancing tolerability and minimizing the likelihood of deleterious effects, particularly to improve recommendations for older adults.

In contrast to the levels of discomfort during exercise, DOMS was significantly higher (p=0.034) in the HIRT group compared to the BFRT group when measured 24 h after training (except after the first exercise session). However, it should be noted that VAS scores were not extremely elevated after exercise bouts and were very low on average throughout the whole training period (first session excluded). The reported level of DOMS was slightly more accentuated after EOE sessions. A difference in DOMS between BFRT and HIRT training is consistent with findings of previous studies, demonstrating that BFRT results in reduced muscle damage due to a decrease in mechanical stress, while the training stimulus depends largely on metabolic stress (Pearson and Hussain 2015). Notably, while our study was designed somewhat similar to Margaritelis et al. 2015, who managed to overcome the RBE in young adults, the observed decrease in DOMS levels can also be explained as a RBE, which helps the body adapt to training and reduces muscle damage such as often seen after HIRT (McHugh 2003). The assumption that also BFRT can evoke a RBE is supported by the findings of a study conducted by Sieljacks et al., (2016), who found that repeated exercise during a program of BFRT performed until failure of willpower induced a protective effect against muscle damage, increased activity and faster recovery of muscle strength. However, in contrast to our study, the study of Sieljacks et al. (2016) was conducted in young men. Further research should investigate how RBE following BFRT play out in an older population, taking into account possible age-related differences in muscle adaptation and recovery.

We designed an exercise protocol with repeated sessions of EOE somewhat similar to the study undertaken by Margaritelis et al. (2015) which demonstrated that the alternation of eccentric-only and concentric-only exercises has the capacity to entirely eliminate RBE. The authors posit that concentric exercise leads to a reduction in the number of sarcomeres, an increase in the susceptibility of the muscle to damage, and that the cessation of eccentric exercise after a period of recovery negates the adaptation effect. The protocol utilised in the present study was founded upon the same principle yet differed in that the older men engaged in a total of three sessions of traditional RE, whereas in the study by (Margaritelis et al. 2015), concentric-only exercise was performed over the course of one month in young adults. This discrepancy, which was chosen in order to simulate repeated muscle damaging bouts in a limited number of weeks in older adults, may have caused that we were unable to achieve a similar effect. This shows that older adults are still able to benefit from the RBE, even when switching between traditional RE and EOE sessions. Notably, a limitation in this study was that we did not include measurements of CK and DOMS before each training session.

High-intensity resistance training (HIRT), particularly eccentric training, is often discouraged in older adults due to concerns regarding potential muscle injury, delayed recovery from such damage, and thus a risk of adverse effects rather than training-induced improvements. However, repetitive eccentric training has been demonstrated to promote muscular adaptations, resulting in a RBE that not only enhances resistance to injury but also improves overall recovery (Lovering and Brooks 2014). Despite that in our study participants switched between EOE and RE, HIRT still resulted in significant improvements in 1RM strength across all exercises. While participants in both exercise groups (HIRT and BFRT) showed significant strength gains over time (all p ≤ 0.001), HIRT proved to be more effective than BFRT in increasing 1RM in leg extension (p=0.023) and leg press (p=0.007). This suggests that this training modality provides a potent stimulus for muscular strength development in older adults. However, it is important to consider that the participants included in this study were relatively fit, albeit unaccustomed to RE. Therefore, our findings may not necessarily translate to individuals with frailty or functional impairments. It is recommended that the application of HIRT in older adults is supervised by professionals or reserved for older adults with training experience who then gradually increase their intensity levels.

A potential limitation of this study is the exclusion of female participants, which limits the generalisability of the findings to a broader population. Future research should explore whether the observed effects of HIRT and BFRT differ between sexes, as physiological and perceptual responses to RT may vary. Additionally, the BFRT protocol in this study was applied at 80% AOP, this may have resulted in increased discomfort, as evidenced by elevated RPE scores and a higher number of incomplete sets, potentially impacting training adherence and overall tolerance of the BFRT protocol. Finally, although V% was used to standardize and compare the prescribed training volume between participants and groups, this does not mean that the relative training stimulus was equivalent. Because BFRT and HIRT differ in the mechanisms underlying their acute mechanical and physiological demands, a comparable external training volume may represent a different relative stimulus in each group.

## Conclusion

This study demonstrates that both BFRT (with occlusion pressure progressively increased up to 80% ALOP) and HIRT are effective yet demanding training modalities for older adults. BFRT and HIRT appear to differ in their training stimuli, with BFRT being associated with higher metabolic stress and HIRT with higher mechanical load. Notably, the BFRT group experienced higher RPE and lactate levels, resulting in a greater number of incomplete training sets. In contrast, HIRT, despite the application of high loads and greater DOMS, was associated with fewer incomplete sets and lower RPE scores. The RBE was found in both BFRT and HIRT groups. Participants in the HIRT program, incorporating EOE and RE sessions in a 1:3 ratio, exhibited greater 1RM improvements compared to the BFRT group, particularly in the leg extension and leg press exercises. Both training methods should be administered under professional supervision and tailored to the individual’s physical characteristics and health needs of older adults.

## Supplementary Information

Below is the link to the electronic supplementary material.

Supplementary file1 (DOCX 366 KB)

## Abbreviations

1 RM: One repetition maximum
ALOP: Arterial limb occlusion pressure
AOP: Arterial occlusion pressure
BFRT: Blood flow restriction training
BMI: Body mass index
CK: Creatine kinase
RE: Resistent exercise
DOMS: Delayed onset muscle soreness
EOE: Eccentric-only exercises
EWGSOP: European Working Group on Sarcopenia in older people
HIRT: High-intensity resistance training
RBE: Repeated bout effect
RPE: Ratings of perceived exertion
VAS: Visual analogue 10-point scale
