# Supplementary material for: Comparing high-intensity resistance training incorporating periodic eccentric-only exercise with blood flow restriction training in older men: physiological responses and exercise feasibility
Source: Eur J Appl Physiol. 2026 Sep 24;126(11):6139–50. doi: 10.1007/s00421-026-06452-x (PMC13751698; doi:10.1007/s00421-026-06452-x)
Supplement: Supplementary file 1 [file 421_2026_6452_MOESM1_ESM.docx]

**Supplementary Materials**

**Blood-flow restriction versus high-intensity resistance exercise training in older men: A three-group randomized controlled trial**

**Supplemental Figure S1.** CONSORT 2010 Flow Diagram

**Supplemental Table S1. Overview of training protocols and intervention design**

| **Panel A. Training protocols** | | | | | |
| --- | --- | --- | --- | --- | --- |
| **Protocol** | **Intensity (% 1RM)** | **N° exercises** | **Rest Sets** | **Rest Exercises** | **Volume %** |
| BFRT: 4x 12reps/exercise | 40% | 3 | 30” | 3’ | 57.6 |
| RE: 4x 6reps/exercise | 80% | 3 | 2’ | 3’ | 57.6 |
| EOE: 4x 4reps/exercise | 120% | 3 | 2’ | 3’ | 57.6 |
| **Panel B. Weekly intervention schedule** **Week** \| **Blood flow restriction training** \| \| \| \| **High-intensity resistance training** \| \|; \| **1^st^ visit** \| **2^nd^ visit** \| **3^rd^ visit** \| \| **1^st^ visit** \| **2^nd^ visit** \| **3^rd^ visit**; -1 \| 1RM \| \| \| \| 1RM \| \|; 00 \| Blood+BFRT \| Blood \| \| \| Blood+EOE \| Blood \|; 01 \| BFRT \| BFRT \| \| \| RE \| RE \|; 02 \| BFRT \| BFRT \| \| \| RE \| RE \|; 03 \| BFRT \| BFRT \| \| 1RM \| RE \| RE \| 1RM; 04 \| BFRT \| Blood+BFRT \| \| \| EOE \| Blood+RE \|; 05 \| BFRT \| BFRT \| \| \| RE \| RE \|; 06 \| BFRT \| BFRT \| \| \| RE \| RE \|; 07 \| BFRT \| BFRT \| \| 1RM \| RE \| RE \| 1RM; 08 \| BFRT \| Blood+BFRT \| \| \| EOE \| Blood+RE \|; 09 \| BFRT \| BFRT \| \| \| RE \| RE \|; 10 \| BFRT \| BFRT \| \| \| RE \| RE \|; 11 \| BFRT \| BFRT \| \| \| RE \| RE \|; 12 \| BFRT \| Blood \| \| 1RM \| EOE \| Blood \| 1RM; Blood biomarkers were monitored in the morning before the first exercise session of the program and in the morning 48 hours after intervention. In week 4 and 8, the next training session followed immediately after blood collection. 1RM tests were performed on non-training days later in the week and the weight was adjusted accordingly from the subsequent week. The last 1RM test was performed in the week of the last training session or the week after. Abbreviations: 1RM, one-repetition maximum test; BFRT, blood-flow restriction training; RE, traditional resistance exercise; EOE, eccentric-only exercise; Rest Exercises, rest between exercises; Rest Sets, rest between sets. \| \| \| \| \| \| \| | | | | | |

**Supplemental Figure S2.** Overview of exercises

Panel A shows pictures of the eccentric-only exercise (EOE) condition, where the concentric phase of the movement is supported by two coaches. During traditional resistance training (RE) the support of the coaches was absent, but the exercises remained the same. Panel B shows pictures of the blood-flow restriction training condition.

**Supplemental Figure S3. Changes in creatine kinase (CK) levels over time in the BFRT and HIRT groups.** Panel (a) shows CK levels in the BFRT group, and panel (b) shows CK levels in the HIRT group at baseline (pre), 48 hours, 4 weeks, 8 weeks, and 12 weeks. Individual data points are presented along with group distributions. Time p = 0.001*, Group p = 0.664, Time*Group interaction p = 0.033*.

| **Supplementary Table S2. Repeated measures ANOVA results** | | | | | | | | | | | | | | | |
| --- | --- | --- | --- | --- | --- | --- | --- | --- | --- | --- | --- | --- | --- | --- | --- |
|  | | | | | | Time effect | | Group effect | | Interaction effect | | Post-hoc group analysis | | | |
| Variable | Group | Mean pre | SD pre | Mean post | SD post | p-value Time | pη² Time | p-value Group | pη² Group | p-value Time*Group | pη² Time*Group | 2x2 table | | | |
| CK pre-CK 48h post first session | Total | 1.88 | 1.12 | 4.94 | 6.76 | 0.001* | 0.185 | 0.044* | 0.114 | 0.016* | 0.147 |  | BFRT | HIRT | CON |
|  | BFRT | 1.77 | 1.01 | 4.47 | 3.2 | 0.067 | 0.063 |  |  |  |  | BFRT |  |  |  |
|  | HIRT | 1.71 | 0.99 | 8.16 | 10.63 | <0.001* | 0.254 |  |  |  |  | HIRT | 0.209 |  |  |
|  | CON | 2.19 | 1.36 | 2.09 | 1.24 | 0.944 | 0 |  |  |  |  | CON | 0.641 | 0.038* |  |
| CK pre- mean CK 48h after sessions at 4,8,12w | Total | 1.88 | 1.12 | 2.7 | 1.88 | 0.001* | 0.205 | 0.664 | 0.733 | 0.033* | 0.135 |  | BFRT | HIRT | CON |
|  | BFRT | 1.77 | 1.01 | 2.47 | 1.65 | 0.056 | 0.076 |  |  |  |  | BFRT |  |  |  |
|  | HIRT | 1.71 | 0.99 | 3.1 | 2.41 | <0.001* | 0.273 |  |  |  |  | HIRT | 0.661 |  |  |
|  | CON | 2.19 | 1.36 | 2.52 | 1.41 | 0.903 | 0 |  |  |  |  | CON | 0.801 | 0.982 |  |
| Lactate pre-post first session | Total | 2.16 | 1.02 | 4.76 | 3.15 | <0.001* | 0.502 | <0.001* | 0.346 | <0.001* | 0.379 |  | BFRT | HIRT | CON |
|  | BFRT | 2.22 | 1.06 | 7.08 | 2.81 | <0.001* | 0.585 |  |  |  |  | BFRT |  |  |  |
|  | HIRT | 2.14 | 1.09 | 4.55 | 3.09 | <0.001* | 0.239 |  |  |  |  | HIRT | 0.015* |  |  |
|  | CON | 2.1 | 0.95 | 2.27 | 0.82 | 0.781 | 0.001 |  |  |  |  | CON | <0.001* | 0.043* |  |
| 1RM leg extension pre - after 12 weeks | Total | 71.57 | 16.74 | 85.9 | 18.85 | <0.001* | 0.534 | 0.107 | 0.097 | <0.001 | 0.368 |  | BFRT | HIRT | CON |
|  | BFRT | 72.34 | 14.25 | 84.54 | 16.69 | 0.001* | 0.227 |  |  |  |  | BFRT |  |  |  |
|  | HIRT | 71.02 | 18.99 | 98.28 | 15.6 | <0.001* | 0.594 |  |  |  |  | HIRT | 0.49 |  |  |
|  | CON | 71.33 | 17.78 | 74.13 | 16.81 | 0.429 | 0.014 |  |  |  |  | CON | 0.557 | 0.088 |  |
| 1RM leg curl pre - after 12 weeks | Total | 44.43 | 11.07 | 51.3 | 9.1 | <0.001* | 0.391 | 0.504 | 0.03 | 0.002* | 0.247 |  | BFRT | HIRT | CON |
|  | BFRT | 42.21 | 9.51 | 52 | 7.15 | <0.001* | 0.316 |  |  |  |  | BFRT |  |  |  |
|  | HIRT | 44.71 | 10.08 | 55.07 | 7.71 | <0.001* | 0.355 |  |  |  |  | HIRT | 0.649 |  |  |
|  | CON | 46.47 | 13.71 | 46.3 | 10.53 | 0.94 | 0 |  |  |  |  | CON | 0.973 | 0.517 |  |
| 1RM leg press pre - after 12 weeks | Total | 191.54 | 41.74 | 237.3 | 62.84 | <0.001* | 0.605 | 0.001* | 0.274 | <0.001* | 0.574 |  | BFRT | HIRT | CON |
|  | BFRT | 170.74 | 33.31 | 238.89 | 52.61 | <0.001* | 0.55 |  |  |  |  | BFRT |  |  |  |
|  | HIRT | 206.21 | 41.17 | 286.31 | 39.38 | <0.001* | 0.628 |  |  |  |  | HIRT | 0.013* |  |  |
|  | CON | 198.07 | 43.95 | 183.33 | 49.81 | 0.132 | 0.051 |  |  |  |  | CON | 0.587 | 0.001* |  |
